# Paclitaxel-loaded nanoparticles of star-shaped cholic acid-core PLA-TPGS copolymer for breast cancer treatment

**DOI:** 10.1186/1556-276X-8-420

**Published:** 2013-10-17

**Authors:** Xiaolong Tang, Shuyu Cai, Rongbo Zhang, Peng Liu, Hongbo Chen, Yi Zheng, Leilei Sun

**Affiliations:** 1 Stem Cell Engineering and Technology Research Center, School of Medicine, Anhui University of Science and Technology, Huainan 232001, China; 2 Nankai Hospital, Nankai Clinical School, Tianjin Medical University, Tianjin 300100, China; 3 The Shenzhen Key Laboratory of Gene and Antibody Therapy, Division of Life Sciences, Graduate School at Shenzhen, Tsinghua University, Shenzhen 518055, China; 4 Northeastern University, Boston, MA 02115, USA

**Keywords:** Paclitaxel, Breast cancer, Nanoparticles, Drug delivery, Star-shaped copolymer

## Abstract

A system of novel nanoparticles of star-shaped cholic acid-core polylactide-d-α-tocopheryl polyethylene glycol 1000 succinate (CA-PLA-TPGS) block copolymer was developed for paclitaxel delivery for breast cancer treatment, which demonstrated superior *in vitro* and *in vivo* performance in comparison with paclitaxel-loaded poly(d,l-lactide-*co*-glycolide) (PLGA) nanoparticles and linear PLA-TPGS nanoparticles. The paclitaxel- or couramin 6-loaded nanoparticles were fabricated by a modified nanoprecipitation method and then characterized in terms of size, surface charge, surface morphology, drug encapsulation efficiency, and *in vitro* drug release. The CA-PLA-TPGS nanoparticles were found to be spherical in shape with an average size of around 120 nm. The nanoparticles were found to be stable, showing no change in the particle size and surface charge during 90-day storage of the aqueous solution. The release profiles of the paclitaxel-loaded nanoparticles exhibited typically biphasic release patterns. The results also showed that the CA-PLA-TPGS nanoparticles have higher antitumor efficacy than the PLA-TPGS nanoparticles and PLGA nanoparticles *in vitro* and *in vivo*. In conclusion, such nanoparticles of star-shaped cholic acid-core PLA-TPGS block copolymer could be considered as a potentially promising and effective strategy for breast cancer treatment.

## Background

Cancer remains a major public health problem worldwide [1]. The three most commonly diagnosed types of cancer among women in 2012 were that of the breast, lung and bronchus, and colorectum, accounting for about half of the estimated cancer cases in women [1]. However, current treatment options for breast cancer are still limited mainly to surgical resection, chemotherapy, and radiotherapy, which are highly aggressive and/or nonspecific and often accompanied with undesirable and potentially serious side effects because anticancer drugs also exert excessive toxicity to healthy tissues and cells [2,3]. Nanomedicine, especially drug formulation by polymeric nanoparticles, has shown a great deal of promise to provide solutions to such problems in cancer treatment [4,5]. In recent years, a lot of attention has been paid to the biodegradable polymeric nanoparticles for their passive and active drug targeting to the desired sites after various routes of administration [6,7]. In addition, the nanoparticles used as drug carriers possess other advantages including a stable structure, high entrapment efficiency, high cellular uptake, more desirable biodistribution, and more reasonable pharmacokinetics as well as preferentially accumulate at the tumor site through the enhanced permeability and retention effect [8,9]. Polymeric nanoparticles were also found to reduce or overcome drug resistance of tumor cells [10].

Biodegradable polymers have great application potential in biomedical fields including drug delivery and tissue engineering. Among them, the polyester family including poly(d,l-lactide-*co*-glycolide) (PLGA), polylactide (PLA), and polyglycolide (PGA) is most extensively investigated due to its good biocompatibility and biodegradability [9,11]. Despite the well-established importance, this kind of polymers still has limitations in particular applications. It is well known that the autocatalytic effect and the acidic degradation products of these polyesters cause unfavorable effects. In addition, the degradation rate of polyesters such as PLA and PLGA is too slow due to their hydrophobic nature to meet the therapeutic needs [12,13]. It was also reported that PLA- and PLGA-based nanoparticles can be rapidly cleared in the liver and captured by the reticuloendothelial system (RES) when they are administrated into the blood circulation [14,15]. These drawbacks could be overcome by the introduction of d-α-tocopheryl polyethylene glycol 1000 succinate (TPGS) into the hydrophobic PLA backbone [16]. TPGS, a water-soluble derivative of the natural form of d-α-tocopherol, is formed by esterification of vitamin E succinate with poly(ethylene glycol) (PEG) 1000. It was found that TPGS could improve the aqueous solubility of drugs including taxanes, antibiotics, cyclosporines, and steroids. In addition, TPGS could serve as an excellent molecular biomaterial for overcoming multidrug resistance and as an inhibitor of P-glycoprotein to increase the cytotoxicity and oral bioavailability of antitumor agents [17].

Though PLGA-based nanoparticles and PLA-TPGS-based nanoparticles have been extensively studied as delivery vehicles of drugs, most of them were focused on making use of linear polymers. In recent years, branched polymers, such as hyper-branched polymers, star-shaped polymers, and dendrimers, have obtained great attention due to their useful mechanical and rheological properties [9,18,19]. A star-shaped block polymer is a branched polymer molecule in which a single branch point (core) gives rise to multiple linear chains or arms [20]. In comparison with linear polymers at the same molar mass, nanocarriers based on a star-shaped polymer molecular structure showed a smaller hydrodynamic radius, lower solution viscosity, higher drug content, and higher drug entrapment efficiency [21,22]. Therefore, in this research, novel delivery systems of star-shaped block copolymers based on PLA and TPGS with unique architectures were developed, which would provide valuable insights for fabricating ideal and useful drug carriers for nanomedicine applications [23,24]. Cholic acid (CA) is one of the two major bile acids produced by the liver where it is synthesized from cholesterol. It is composed of a steroid unit with one carboxyl group and three hydroxyl groups. CA was chosen as the polyhydroxy initiator due to its biological origin, which may obtain better biocompatibility for polymers incorporated with the CA moiety [25]. Moreover, it was reported that CA-functionalized star-shaped polymers could exhibit faster hydrolytic degradation rates in comparison with linear homopolymers such as PLA and poly(ϵ-caprolactone) (PCL). The existence of the CA moiety in biomaterials could also significantly increase both cell adherence and proliferation [26].

In this research, the star-shaped block copolymer CA-PLA-TPGS with three branch arms was used for developing a superior nanocarrier of anticancer agents with satisfactory drug content and entrapment efficiency for breast cancer treatment. The star-shaped CA-PLA-TPGS nanoparticles containing paclitaxel (PTX) as a model drug were characterized, and the anticancer effect of nanoparticles was evaluated both *in vitro* and *in vivo*.

## Methods

### Materials

TPGS, 4′-6′-diamino-2-phenylindole (DAPI), and PLA (*M*_w_ approximately 25,000) were purchased from Sigma-Aldrich (St. Louis, MO, USA). CA-PLA-TPGS copolymer (*M*_w_ approximately 23,000) and PLA-TPGS (*M*_w_ approximately 23,000) copolymer were obtained from the Graduate School at Shenzhen, Tsinghua University. PTX was provided by Beijing Union Pharmaceutical Factory (Beijing, China). All chromatographic solvents were of high-performance liquid chromatography (HPLC)-grade quality, and all other chemicals used were of the highest grade commercially available. Human breast adenocarcinoma cell line MCF-7 was obtained from American Type Culture Collection (ATCC; Rockville, MD, USA).

### Characterization of CA-PLA-TPGS copolymers

Proton nuclear magnetic resonance (^1^H NMR; Bruker AMX 500, Madison, WI, USA) was applied to confirm the structure of the synthesized CA-PLA-TPGS copolymer. Fourier transform infrared (FTIR) spectrophotometry (Thermo Nicolet, Madison, WI, USA) was further applied to investigate the molecular structure of the CA-PLA-TPGS copolymer. In brief, the samples for FTIR analysis were prepared by grinding 99% KBr with 1% CA-PLA-TPGS copolymer and then pressing the mixture into a transparent tablet.

### Fabrication of PTX-loaded nanoparticles

A modified nanoprecipitation method was used to entrap PTX into the CA-PLA-TPGS nanoparticles (NPs) [9]. Briefly, a pre-weighed amount of drug powder and 100 mg of CA-PLA-TPGS copolymer were dissolved in 8 mL of acetone by vortexing and sonication. This mixture was dropwise added into 100 mL of 0.03% TPGS aqueous solution under stirring. The resulting nanoparticle suspension was then stirred at room temperature overnight to remove acetone completely. The nanoparticle suspension was centrifuged at 25,000 rpm for 15 min and then washed two to three times to remove the emulsifier and unloaded drug. In the end, the dispersion was lyophilized for 48 h for further use. PTX-loaded PLGA nanoparticles and PLA-TPGS nanoparticles and coumarin 6-loaded CA-PLA-TPGS NPs were fabricated in a similar manner. The lyophilized nanoparticles were redispersed in phosphate buffer solution (PBS) before use.

### Characterization of PTX-loaded nanoparticles

#### Size, surface charge, and morphology of the nanoparticles

The nanoparticle size and zeta potential were determined using Malvern Mastersizer 2000 (Zetasizer Nano ZS90, Malvern Instruments Ltd., Malvern, UK). Before measurement, the freshly fabricated nanoparticles were appropriately diluted. All measurements were measured at room temperature after equilibration for 10 min. The data were obtained with the average of three measurements.

The surface morphology of nanoparticles was examined by field emission scanning electron microscopy (FESEM, JEOL JSM-6301F, Tokyo, Japan). To prepare samples for FESEM, the nanoparticles were fixed on the stub using a double-sided sticky tape and then coated with a platinum layer using a JFC-1300 automatic fine platinum coater (JEOL, Tokyo, Japan) for 40 s.

#### Drug content and entrapment efficiency

To determine the contents of drug loading (LC) and entrapment efficiency (EE) of the PTX-loaded nanoparticles, a predetermined amount of nanoparticles was dissolved in 1 mL methylene dichloride under vigorous vortexing. The solution was transferred to 5 mL of mobile phase consisting of acetonitrile and deionized water (50:50, *v*/*v*). A nitrogen stream was introduced to evaporate the methylene dichloride for approximately 20 min, and then a clear solution was obtained for HPLC analysis (LC 1200, Agilent Technologies, Santa Clara, CA, USA). A reverse-phase C_18_ column (250 × 4.6 mm, 5 μm, Agilent Technologies, Santa Clara, CA, USA) was used at 25°C. The flow rate of the mobile phase was 1 mL/min. The column effluent was detected using a UV detector at *λ*_max_ of 227 nm. The measurement was performed in triplicate. The LC and EE of the PTX-loaded nanoparticles were calculated by the following equations, respectively:

$$\text{LC}\;\left(\text{\%}\right)=\frac{\text{Weight}\;\text{of}\;\text{PTX}\;\text{in}\;\text{the}\;\text{nanoparticles}}{\text{Weight}\;\text{of}\;\text{the}\;\text{nanoparticles}}\times 100\text{\%},$$

$$\text{EE}\;\left(\text{\%}\right)=\frac{\text{Weight}\;\text{of}\;\text{PTX}\;\text{in}\;\text{the}\;\text{nanoparticles}}{\text{Weight}\;\text{of}\;\text{the}\;\text{feeding}\;\text{PTX}}\times 100\text{\%}.$$

#### In vitro drug release assay

*In vitro* PTX release from nanoparticle formulations was performed as described previously. In brief, 5 mg of accurately weighted lyophilized nanoparticles was put into a centrifuge tube and redispersed in 8 mL PBS (containing 0.1% *w*/*v* Tween 80, pH 7.4). The tube was put into an orbital shaker water bath and vibrated at 130 rpm at 37°C. At certain time intervals, the tube was taken out and centrifuged at 25,000 rpm for 15 min. The supernatant was then transferred into a glass test tube for HPLC analysis. The pellet was resuspended in 8 mL fresh PBS and put back into the shaker bath for subsequent determination. The accumulative release of PTX from nanoparticles was plotted against time.

### Cellular uptake of nanoparticles

In this research, coumarin 6 served as a model fluorescent molecule, which can be entrapped in the linear PLGA nanoparticles, linear PLA-TPGS nanoparticles, and star-shaped CA-PLA-TPGS nanoparticles for qualitative and quantitative studies on cellular uptake by tumor cells such as MCF-7 cells. MCF-7 cells were cultured in Dulbecco's modified essential medium (DMEM) supplemented with 10% heat-inactivated fetal bovine serum and antibiotics. The culture was kept in 95% air humidified atmosphere containing 5% CO_2_ at 37°C. The cells were incubated with 250 μg/mL coumarin 6-loaded CA-PLA-TPGS nanoparticles at 37°C for 2 h, rinsed with cold PBS three times, and then fixed by methanol for 25 min. Cells were stained with DAPI for 30 min to display the nuclei and rinsed twice with PBS.

The MCF-7 cells were observed by confocal laser scanning microscopy (CLSM; LSM 410, Zeiss, Jena, Germany) with an imaging software. The images of the cells were determined with a differential interference contrast channel, and the images of coumarin 6-loaded nanoparticles and the nuclei of the cells stained by DAPI were recorded with the following channels: a blue channel (DAPI) with excitation at 340 nm and a green channel (coumarin 6) with excitation at 485 nm [27,28].

For the quantitative studies, MCF-7 cells at the density of 1 × 10^4^ cells/well were plated in 96-well plates and kept overnight. The cells were equilibrated with Hank's buffered salt solution (HBSS) at 37°C for 60 min before coumarin 6-loaded nanoparticles were added at concentrations of 100, 250, and 500 μg/mL. After incubation for 2 h, the medium was removed and the wells were rinsed three times with 50 μL cold PBS. Finally, 50 μL of 0.5% Triton X-100 in 0.2 N sodium hydroxide was put into each sample well to lyse the cells.

### *In vitro* cytotoxicity of PTX-loaded nanoparticles

MCF-7 cells were seeded in 96-well plates at the density of 5 × 10^3^ viable cells per well in 100 μl of culture medium and incubated overnight. The cells were incubated with the PTX-loaded CA-PLA-TPGS nanoparticles, PLA-TPGS nanoparticle suspension, and Taxol^®^ at equivalent drug concentrations ranging from 0.25 to 25 μg/mL or the placebo CA-PLA-TPGS nanoparticles of the same particle concentration for 24, 48, and 72 h. At certain time intervals, the nanoparticles were replaced with DMEM containing (3-(4,5-dimethylthiazol-2-yl)-2,5-diphenyltetrazolium bromide (MTT; 5 mg/mL), and cells were then incubated for additional 4 h. MTT was aspirated off and DMSO was added to each well to solubilize the formazan crystals formed in viable cells. Absorbance was recorded at 570-nm wavelength using a 96-well microplate reader. Untreated cells were considered as a negative control with 100% viability, and cells without addition of MTT were performed as blank to calibrate the spectrophotometer to zero absorbance. The half maximal inhibitory concentration (IC_50_), the drug concentration at which cell growth was inhibited by 50% relative to untreated control cells, was calculated by curve fitting of the cell viability versus drug concentration data [29].

### *In vivo* studies

The Administrative Committee on Animal Research in the Anhui University of Science and Technology approved all the protocols for the proposed human breast cancer cell lines and animal experiments. Female severe combined immunodeficient (SCID) mice weighing 15 to 20 g were obtained from the Institute of Laboratory Animal Sciences, Chinese Academy of Medical Science. MCF-7 cancer cells in the medium were inoculated subcutaneously to mice in the amount of 2 × 10^6^ cells per mouse at the right axilla, and the subcutaneous tumor growth in each mouse was monitored. The length and width of tumors were determined using a vernier caliper, and the tumor volume (*V*) was calculated as *V* = *d*^2^ × *D* / 2, where *d* and *D* are the shortest and the longest diameter of the tumor in millimeters, respectively [30]. When the tumor volume reached approximately 50 mm^3^ (set as the 0 day), treatments were performed. The mice were randomly divided into three groups (each group has five mice, *n* = 5). The two formulations of paclitaxel, i.e., the drug-loaded CA-PLA-TPGS nanoparticles and Taxol^®^, were injected intra-tumorally at a single dose of 10 mg PTX/kg in PBS on days 0, 4, and 8. Physiological saline served as control. Mice were sacrificed by decapitation 12 days after treatment. The terminal tumor weight (mg) was determined and applied to evaluate the antitumor effects.

### Statistical methods

All experiments were performed at least three times unless otherwise mentioned. Student's *t* test statistical analysis was carried out with SPSS 17.0 software, with *P* < 0.05 considered to indicate a significant difference.

## Results and discussions

### Characterization of CA-PLA-TPGS copolymers

**Figure 1:**
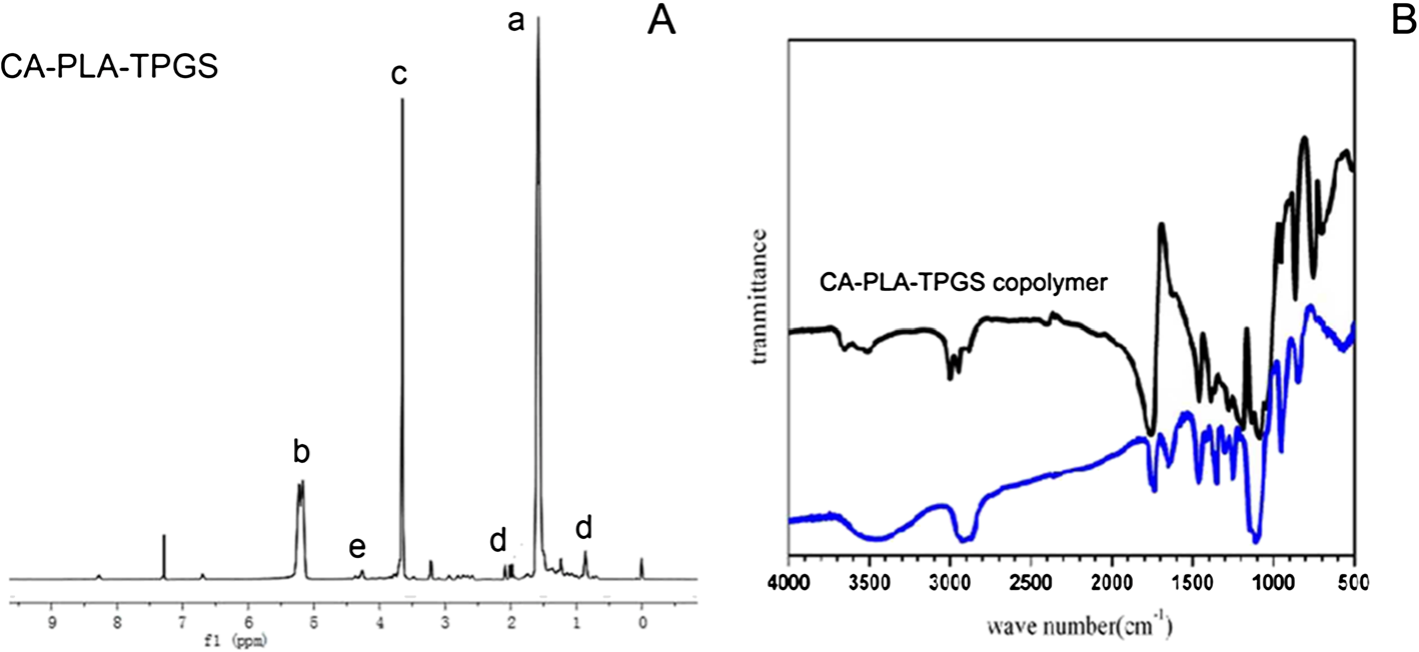
^**1**^**H NMR and FTIR spectra. (A)** Typical ^1^H NMR spectrum of the CA-PLA-TPGS copolymer. **(B)** FTIR spectra of the CA-PLA-TPGS copolymer (black) and TPGS (blue).

In order to confirm the formation of the CA-PLA-TPGS copolymer, ^1^H NMR spectrum is recorded and is shown in Figure 1A. For the CA-functionalized star-shaped polymer CA-PLA-TPGS, the typical signals from CA moiety, TPGS moiety, and LA monomer repeating units can be observed. ^1^H NMR (CDCl_3_): a (*δ* = 1.62 ppm, LA repeating unit: -CHC*H*_3_), b (*δ* = 5.21 ppm, LA repeating unit: **-**C*H*CH_3_), c (*δ* = 3.65 ppm, TPGS repeating unit: -C*H*_2_C*H*_2_O-), d (*δ* = 0.50 to 2.40 ppm, CA moiety: -C*H*_2_- and -C*H*-), e (*δ* = 4.38 ppm, terminal hydroxyl group of CA-PLA: -C*H*OH). Figure 1B shows the FTIR spectra of the CA-PLA-TPGS copolymer and TPGS. The carbonyl band of TPGS appears at 1,730 cm^-1^. For the CA-PLA-TPGS copolymer, the carbonyl band was shifted to 1,755 cm^-1^. Overlapping of the CH stretching band of PLA at 2,945 cm^-1^ and that of TPGS at 2,880 cm^-1^ was observed. The absorption band at 3,400 to 3,650 cm^-1^ is attributed to the terminal hydroxyl group, and that at 1,050 to 1,250 cm^-1^ is due to the C-O stretching. The results confirmed that the CA-PLA-TPGS copolymer was synthesized by ring-opening polymerization.

### Nanoparticle fabrication

PTX-loaded CA-PLA-TPGS nanoparticles were produced by a modified nanoprecipitation method, in which acetone was chosen as an acceptable solvent. Nanoprecipitation could provide a mild, facile, and low energy input method for the fabrication of polymeric nanoparticles [31]. The drug and polymer could be completely dissolved in acetone to form a clear and homogenous solution. After that, the acetone solution was injected into an aqueous solution under stirring to precipitate the water-insoluble PTX instantaneously. Meanwhile, a rapid precipitation of the hydrophobic PLA segment of the star-shaped copolymer occurs, resulting in spontaneous production of PTX-encapsulated CA-PLA-TPGS nanoparticles [9]. A stable dispersion of PTX-loaded nanoparticles was then produced after stirring to remove organic solvent acetone. In the end, the nanoparticles exhibit a core-shell configuration with hydrophobic PLA as the core encapsulating water-insoluble PTX and the TPGS segment as the hydrophilic stabilization shell [9].

### Nanoparticle characterization

#### Size, surface morphology, zeta potential, and entrapment efficiency

**Figure 2:**
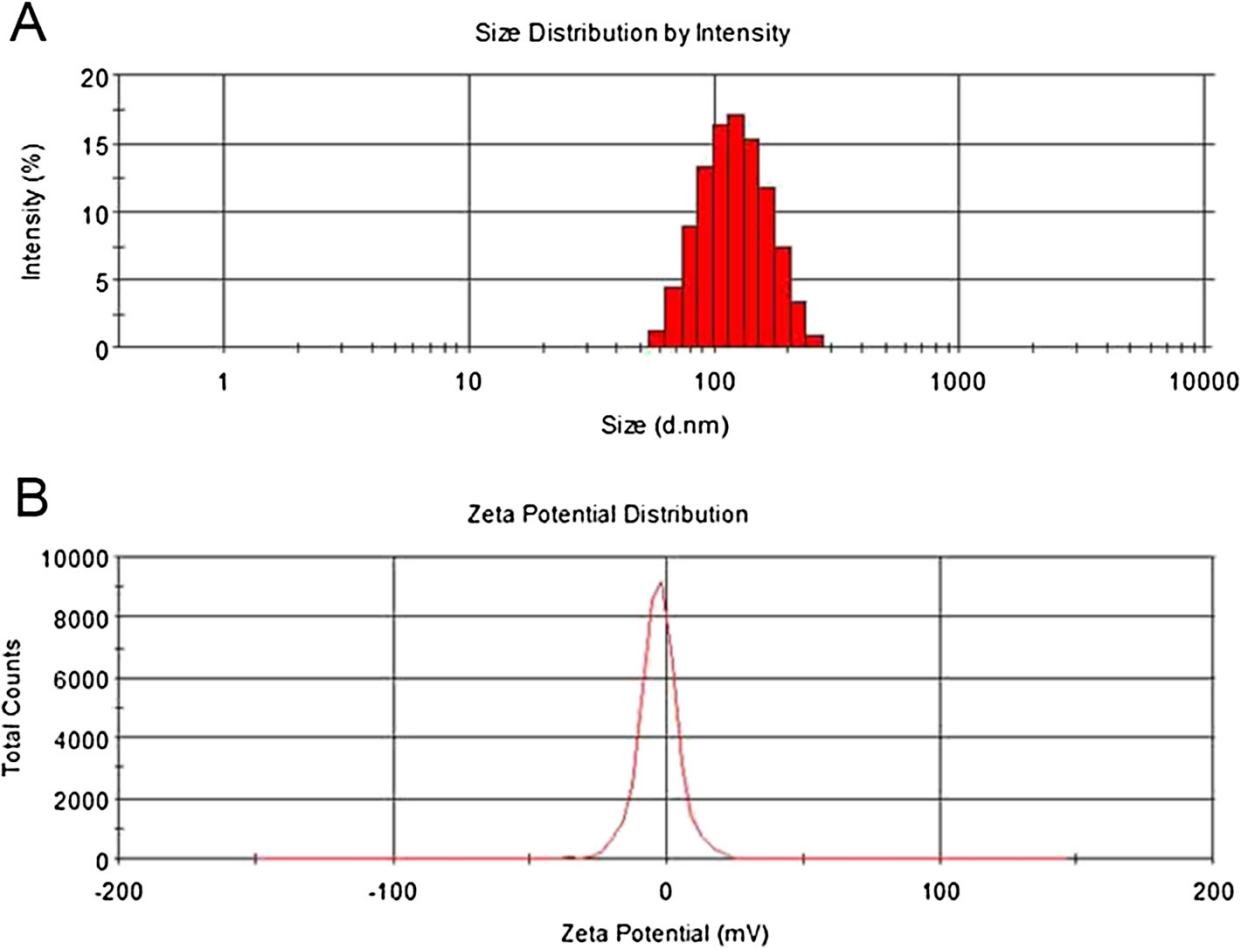
**Size distribution and zeta potential distribution. (A)** Size distribution of the star-shaped CA-PLA-TPGS nanoparticles detected by DLS. **(B)** Zeta potential distribution of the star-shaped CA-PLA-TPGS nanoparticles.

The particle size and size distribution of the PTX-loaded nanoparticles were detected using dynamic light scattering (DLS) equipment, and the data were displayed in Table 1. Particle size and surface properties of the nanoparticles play a crucial role in drug release kinetics, cellular uptake behavior, as well as *in vivo* pharmacokinetics and tissue distribution [32]. The average hydrodynamic size of the PTX-loaded nanoparticles is approximately 110 ~ 140 nm in diameter, which is in the excellent size range for accumulating readily in the tumor vasculature due to enhanced permeation and retention effects [33]. The results revealed that the size of the CA-PLA-TPGS nanoparticles was substantially smaller than that of the PLGA and PLA-TPGS nanoparticles; this was probably due to the star-shaped and constrained geometry architecture of the copolymer. In the present study, both star-shaped CA-PLA-TPGS nanoparticles and linear PLA-TPGS nanoparticles showed a relatively narrow particle size distribution (PDI < 0.20), which makes them particularly suitable for use in drug delivery systems. The size distribution of the PTX-loaded CA-PLA-TPGS nanoparticles obtained from DLS is displayed in Figure 2A.

**Table 1:** Characterization of PTX-loaded nanoparticles

**Polymer**	**Size (nm)**	**PDI**	**ZP (mV)**	**LC (%)**	**EE (%)**
PLGA	134.3 ± 4.8	0.267	-22.8 ± 0.2	8.01	76.39
PLA-TPGS	125.7 ± 3.5	0.195	-19.3 ± 0.4	8.64	84.33
CA-PLA-TPGS	112.9 ± 3.1	0.179	-13.0 ± 0.9	10.05	98.81

PDI, polydispersity index; ZP, zeta potential; LC, loading content; EE, entrapment efficiency; *n* = 3.

**Figure 3:**
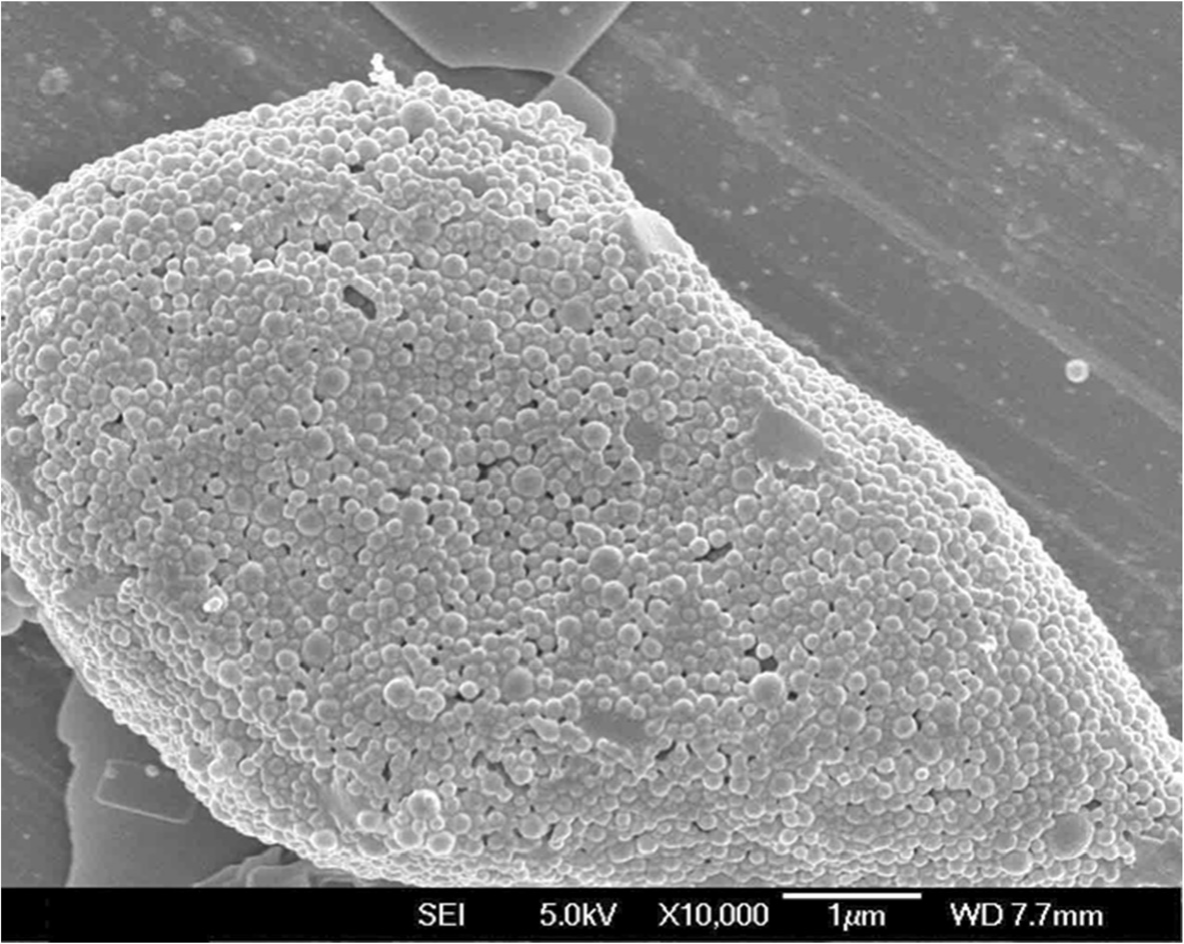
FESEM image of the star-shaped CA-PLA-TPGS nanoparticles.

In an attempt to observe the surface morphology of the nanoparticles, the FESEM study was performed. It can be seen from Figure 3 that all the nanoparticles have a nearly spherical shape and the mean particle size is about 120 nm, which is in agreement with the data from the DLS experiment.

Zeta potential is an important predictor of dispersion stability of nanoparticles. A high absolute value of the zeta potential means high surface charge of the nanoparticles. The zeta potential distribution of the PTX-loaded star-shaped CA-PLA-TPGS nanoparticles is displayed in Figure 2B. As displayed in Table 1, the zeta potential of the PTX-loaded CA-PLA-TPGS nanoparticles and the PLA-TPGS nanoparticles was determined to be -13.0 and -19.3 mV, respectively, which is slightly higher than that of the PLGA nanoparticles of zeta potential about -22.8 mV. The negative surface charge of the nanoparticles may be due to the presence of ionized carboxyl groups of PLA and PGA segments [28].

It can also be found from Table 1 that the contents of drug loading and entrapment efficiency of the CA-PLA-TPGS nanoparticles were higher than those of the PLA-TPGS nanoparticles and the PLGA nanoparticles, indicating the higher binding affinity between the star-shaped core region PLGA and hydrophobic PTX. Moreover, the drug loading content of PTX in the CA-PLA-TPGS nanoparticles could reach approximately 10.0% which is ideal for an efficient drug delivery vehicle. After redispersion in PBS, the mean size and size distribution of the PTX-loaded nanoparticles were nearly not changed during the 3 months of follow-up, suggesting that the PTX-loaded nanoparticles had good stability and redispersion ability.

#### Stability of PTX-loaded nanoparticles

**Figure 4:**
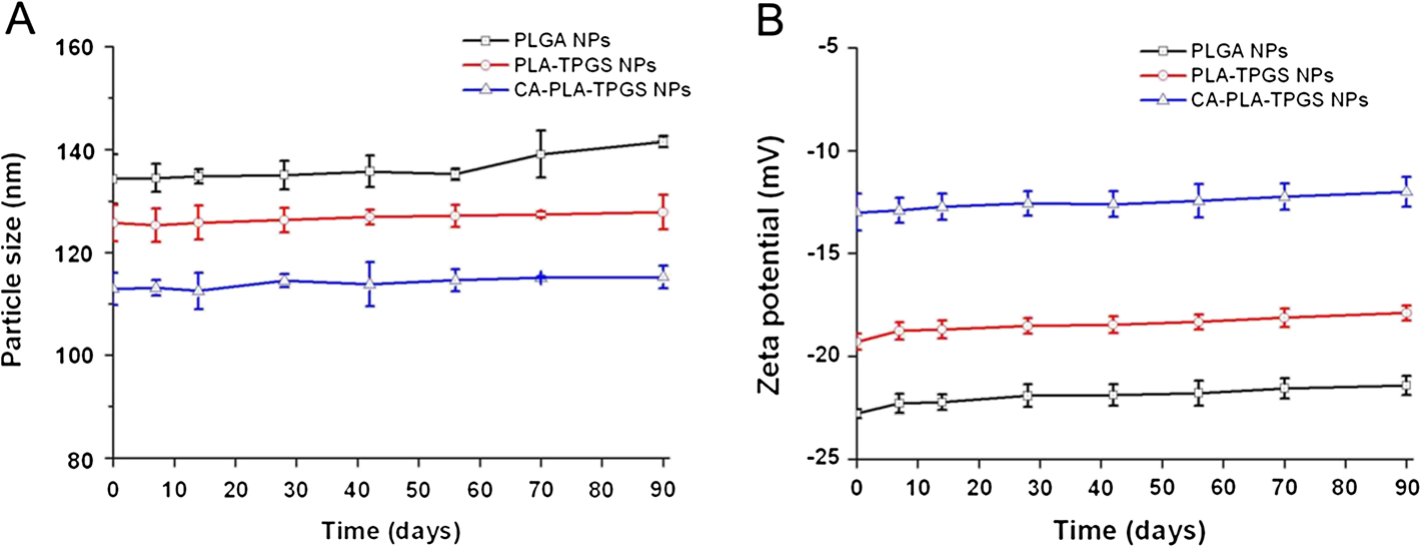
***In vitro *stability of the PTX-loaded nanoparticles. (A)** The size distribution of PTX-loaded PLGA, PLA-TPGS, and CA-PLA-TPGS NPs for 90-day storage at 4°C. **(B)** The zeta potential of PTX-loaded PLGA, PLA-TPGS, and CA-PLA-TPGS NPs for 90-day storage at 4°C.

In biomedical applications, nanoparticles have to be hydrophilic and maintain a superior stability in biological media. Hydrophilic PEG has been the focus of research as an effective coating material for hydrophobic nanoparticles due to its ability to resist protein fouling and provide steric hindrance preventing nanoparticles from aggregation [34]. In this research, TPGS is a water-soluble PEG derivative of the natural form of d-α-tocopherol, which may play an important role in ensuring nanoparticle stability. During the storage of the nanoformulation, the absolute value of the zeta potential usually becomes low and the nanoparticles become aggregated, so the size distribution was uneven and the nanoparticles are not so suitable for therapy as the fresh nanoparticles. Thus, we measure the average size and size distribution and the zeta potential of PTX-loaded CA-PLA-TPGS nanoparticles stored at 4°C at days 7, 14, 28, 42, 56, 70, and 90 after the formulation of the nanoparticles. As shown in Figure 4, the size (Figure 4A) and zeta potential (Figure 4B) were not obviously changed at 4°C after 3-month storage, which means that PTX-loaded CA-PLA-TPGS nanoparticles are very stable.

#### In vitro drug release assay

**Figure 5:**
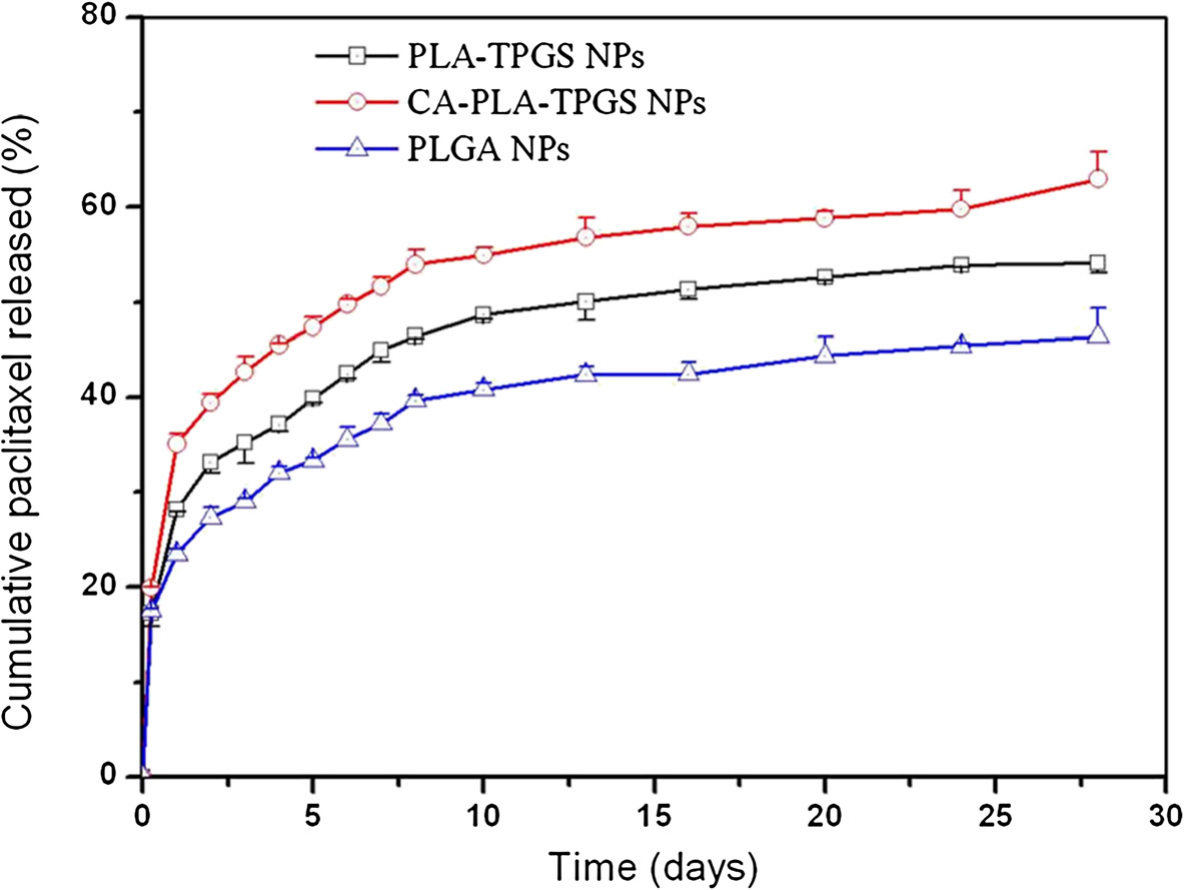
***In vitro *** **release profiles of the PTX-loaded linear PLGA nanoparticles, linear PLA-TPGS nanoparticles, and star-shaped CA-PLA-TPGS nanoparticles.**

The *in vitro* drug release profiles of the PTX-loaded nanoparticles in PBS (containing 0.1% *w*/*v* Tween 80, pH 7.4) in the first 28 days are displayed in Figure 5. Tween 80 was applied to improve the solubility of PTX in the PBS in an attempt to avoid the adhesion of PTX onto the tube wall [35]. The continuous release of drugs from the polymeric nanoparticles could occur either by diffusion of the drug from the polymer matrix or by the erosion of the polymer, which are affected by constituents and architectures of the polymers, surface erosion properties of the nanoparticles, and the physicochemical properties of the drugs [36]. It can be seen from Figure 4 that the release profiles of the PTX-loaded nanoparticles displayed typically biphasic release patterns. The initial burst release in the first 5 days was due to the drug poorly encapsulated in the polymeric core and just located beneath the periphery of the nanoparticles, while the subsequent sustained release was predominantly attributed to the diffusion of the drug, which was well entrapped in the core of nanoparticles. The PTX release from the PLGA nanoparticles, PLA-TPGS nanoparticles, and CA-PLA-TPGS nanoparticles displayed an initial burst of 33.35%, 39.85%, and 47.38% in the first 5 days, respectively. After 28 days, the accumulative PTX release of nanoparticles reached 45% ~ 65%. The accumulative PTX release in the first 28 days was found in the following order: CA-PLA-TPGS nanoparticles > PLA-TPGS nanoparticles > PLGA nanoparticles. The CA-PLA-TPGS nanoparticles displayed the fastest drug release, indicating that the star-shaped CA-PLA-TPGS copolymer was capable of displaying faster drug release than the linear PLA-TPGS nanoparticles when the copolymers had the same molecular weight. In comparison with the linear PLGA nanoparticles, the faster drug release of the PLA-TPGS nanoparticles may be due to the higher hydrophilicity of the TPGS shell, resulting in an easier environment for release medium penetration into the nanoparticle core to make the polymer matrix swell. Similar results can be found in the literature [37,38].

### Cellular uptake of fluorescent CA-PLA-TPGS nanoparticles

**Figure 6:**
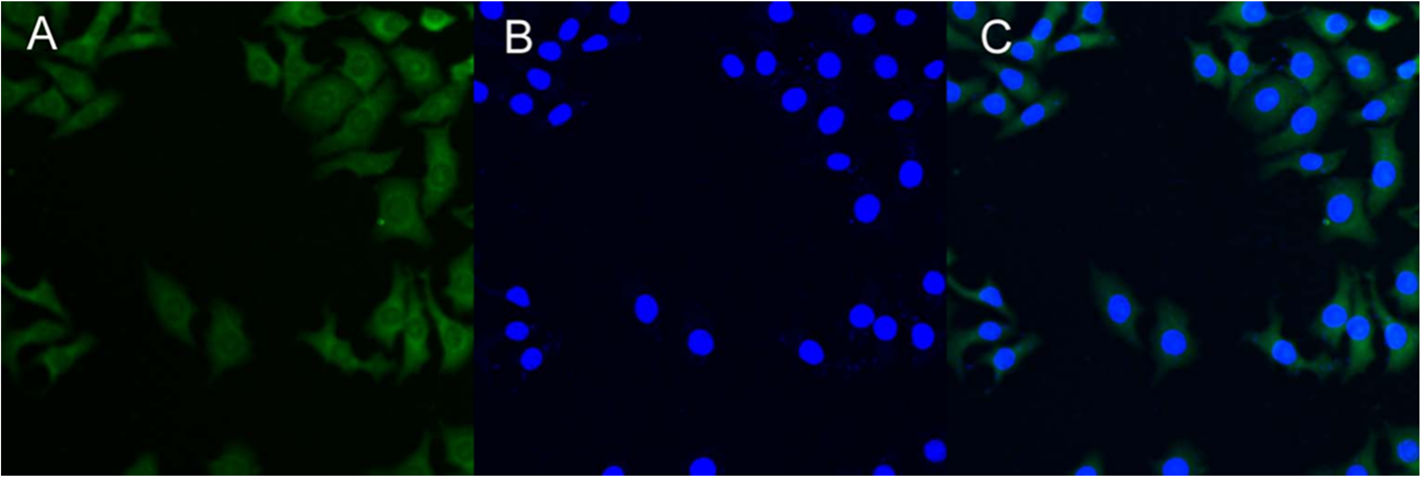
**CLSM images of MCF-7 cells after 4 h of incubation with the coumarin 6-loaded CA-PLA-TPGS nanoparticles.** The coumarin 6-loaded nanoparticles were green, and the cells were stained by DAPI (blue). The cellular uptake was visualized by overlaying images obtained using the EGFP filter and DAPI filter: **(A)** EGFP channel, green; **(B)** DAPI channel, blue; and **(C)** combined EGFP channel and DAPI channel.

The therapeutic effects of the drug-loaded polymeric nanoparticles were dependent on internalization and sustained retention of the nanoparticles by the tumor cells [39]. The *in vitro* studies were capable of providing some circumstantial evidence to show the advantages of the nanoparticle formulation compared with the free drug. Coumarin-6 served as a fluorescent probe in an attempt to represent the drug in the nanoparticles for visualization and quantitative analysis of cellular uptake of the nanoparticles [40]. Figure 6 shows the CLSM images of MCF-7 cells after 24 h of incubation with coumarin 6-loaded CA-PLA-TPGS nanoparticle dispersion in DMEM at the concentration of 250 μg/mL. The images were acquired from (A) the enhanced green fluorescent protein (EGFP) channel (green), (B) the DAPI channel (blue), and (C) the overlay of the two channels. It can be seen from this figure that the coumarin 6-loaded CA-PLA-TPGS nanoparticles (green) were closely located around the nuclei (blue, stained by DAPI), indicating that the fluorescent nanoparticles had been internalized into the MCF-7 cells.

**Figure 7:**
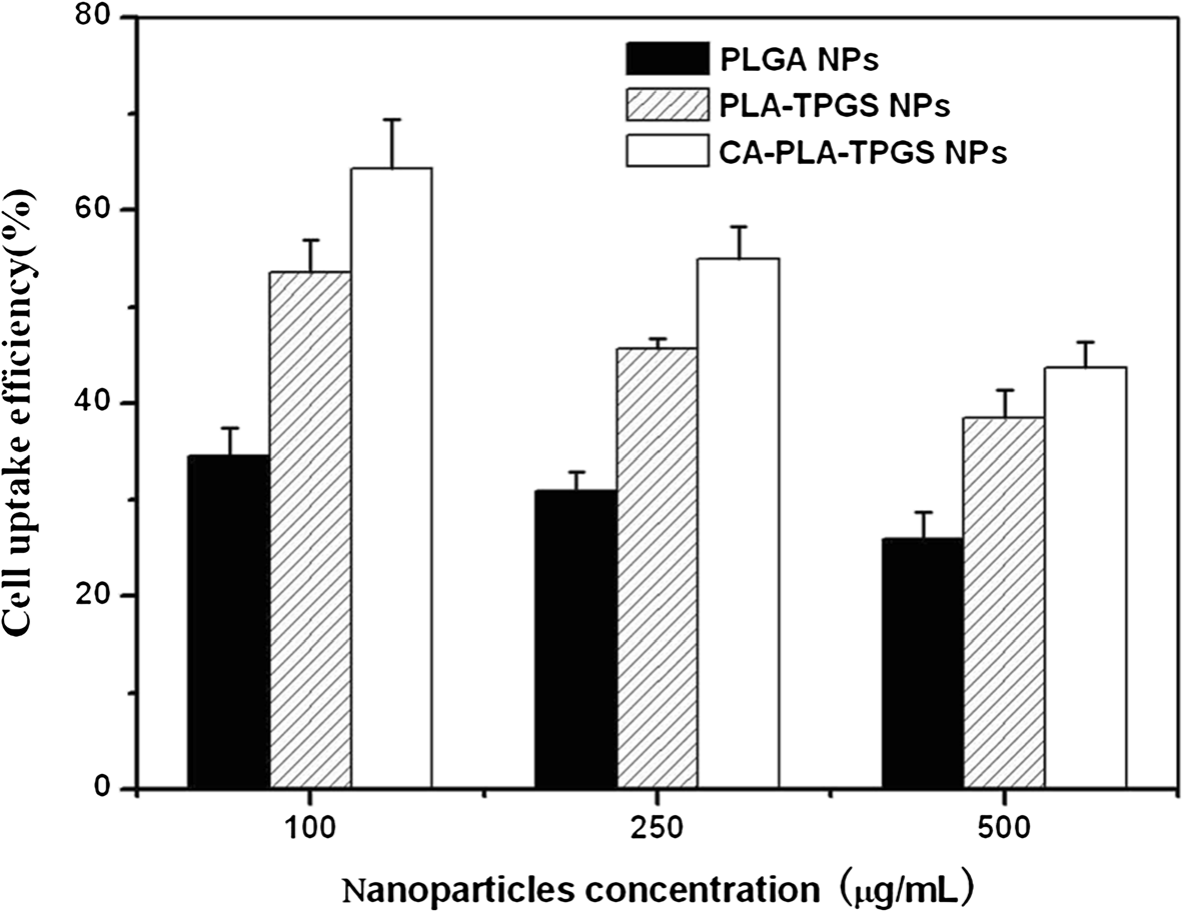
Cellular uptake efficiency of the coumarin 6-loaded nanoparticles.

The cellular uptake efficiency of the coumarin 6-loaded nanoparticles was also measured, and the data are displayed in Figure 7. It can be seen from this picture that the cellular uptake efficiency of all coumarin 6-loaded nanoparticles decreased with the increase of the incubated nanoparticle concentration from 100 to 500 μg/mL. The cellular uptake efficiency of the CA-PLA-TPGS nanoparticles was 1.20-, 1.20-, and 1.14-fold higher than that of the PLA-TPGS nanoparticles at the nanoparticle concentration of 100, 250, and 500 μg/mL, respectively. This may be because of the smaller particle size and increased cell adherence capacity of the CA-PLA-TPGS nanoparticles. The results also showed that the cell uptake efficiency of both the star-shaped CA-PLA-TPGS nanoparticles and the linear PLA-TPGS nanoparticles was higher than that of the linear PLGA nanoparticles. It has been reported in the literature that particle size plays a predominant role in the cellular uptake of biodegradable polymeric nanoparticles [41]. Thus, it can be believed that the CA-PLA-TPGS nanoparticles with smaller particle size would have higher cellular uptake efficiency. Similar results were also obtained by other researchers [42].

### *In vitro* cell viability of PTX-loaded nanoparticles

**Figure 8:**
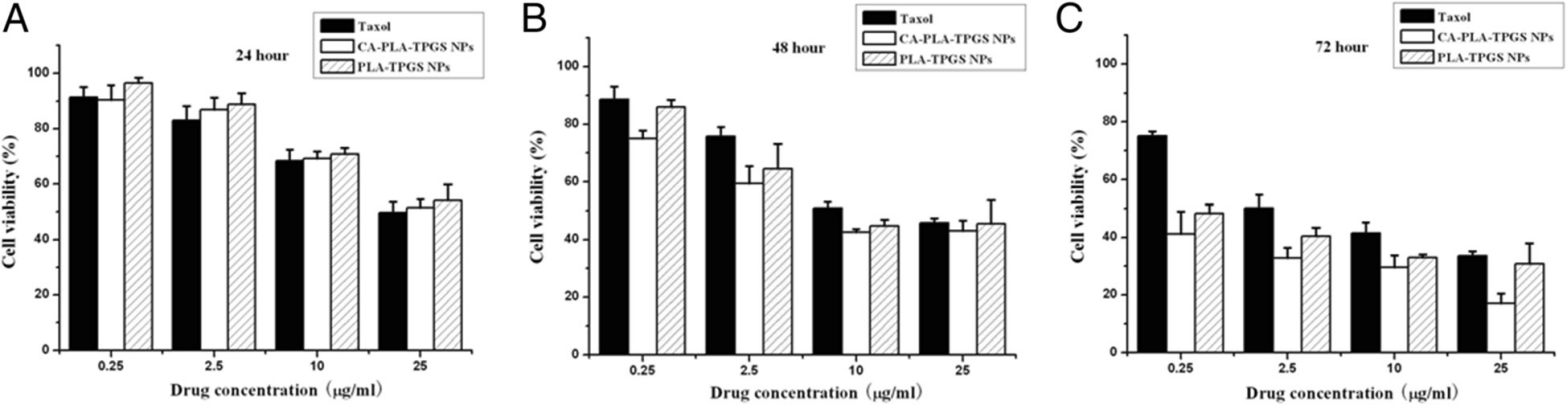
**Cell viability of PTX-loaded nanoparticles compared with that of Taxol^®^ at equivalent PTX dose and nanoparticle concentration. (A)** 24 h. **(B)** 48 h. **(C)** 72 h.

Human MCF-7 cell lines were applied to investigate the cytotoxicity of PTX-loaded nanoparticles. The clinical PTX formulation (Taxol^®^) was designed as the positive control. The different groups of nanoparticles were sterilized using gamma radiation. Figure 8 displays the *in vitro* cell viability of PTX formulated in the linear PLA-TPGS nanoparticles, star-shaped CA-PLA-TPGS nanoparticles, and Taxol^®^ at equivalent PTX concentrations of 0.25, 2.5, 10, and 25 μg/mL. A quantitative colorimetric assay of MTT was used to determine the percentage of viable cells [42]. It can be concluded from Figure 8 that (a) the cell suppression of Taxol^®^ and the drug-loaded polymeric nanoparticles showed both dose- and time-dependent responses. The cell viability decreased steadily with increasing drug dose and incubation time, especially for the drug-loaded star-shaped CA-PLA-TPGS nanoparticles. (b) Though no significant difference in cytotoxicity could be observed among the three formulations after 24 h of incubation, the PTX-loaded star-shaped CA-PLA-TPGS nanoparticles did show much higher cytotoxicity efficacy against MCF-7 cells than the linear PLA-TPGS nanoparticles and Taxol^®^ after 48 and 72 h of incubation. Figure 8 also shows that the MCF-7 cell viability after 24 h of incubation at 10 μg/mL of drug concentration was 68.35% for Taxol^®^, 70.75% for the linear PLA-TPGS nanoparticles, and 69.22% for the star-shaped CA-PLA-TPGS nanoparticles. However, in comparison with the cytotoxicity of Taxol^®^, the MCF-7 cells demonstrated 17.04% and 20.12% higher cytotoxicity for the PTX-loaded star-shaped CA-PLA-TPGS nanoparticles after 48 and 72 h of incubation at the drug concentration of 10 μg/mL, respectively (*P* < 0.05, *n* = 6).

It can also be found that the PTX-loaded star-shaped CA-PLA-TPGS nanoparticles showed increasingly higher therapeutic efficacy for MCF-7 cells than the clinical Taxol^®^ formulation and the linear PLA-TPGS nanoparticles with increasing incubation time. This could be due to the higher cellular uptake and the faster drug release of the PTX-loaded star-shaped CA-PLA-TPGS nanoparticles. The best therapeutic activity in MCF-7 cells was found for the PTX-loaded star-shaped CA-PLA-TPGS nanoparticles at 25 μg/mL of equivalent drug concentration, which could reach as low as 17.09% cell viability after 72 h of incubation. This might be attributed to the enough PTX released from the polymeric nanoparticles and the TPGS component from degradation of the polymer matrix. As we know, TPGS is also cytotoxic and may produce synergistic anticancer effects with PTX [43-45].

The advantages in cancer cell inhibition of the CA-PLA-TPGS nanoparticle formulation > PLA-TPGS nanoparticle formulation > commercial Taxol^®^ formulation could be quantitatively demonstrated in terms of their IC_50_ values, which is defined as the drug inhibitory concentration that is required to cause 50% tumor cell mortality in a designated period. The IC_50_ values of the three PTX formulations of Taxol^®^, the linear PLA-TPGS nanoparticles, and the star-shaped CA-PLA-TPGS nanoparticles on MCF-7 cells after 24, 48, and 72 h of incubation are displayed in Table 2, which are calculated from Figure 8. It can be seen from Table 2 that the IC_50_ value of the PTX-loaded CA-PLA-TPGS nanoparticles on MCF-7 cells was 46.63 μg/mL, which was a degree higher than that of Taxol^®^ after 24 h of incubation. However, the IC_50_ value of Taxol^®^ on MCF-7 cells decreased from 38.13 to 28.32 μg/mL, and that of the PTX-loaded star-shaped CA-PLA-TPGS nanoparticles decreased from 34.71 to 15.22 μg/mL for after 48 and 72 h of incubation, respectively. It can be concluded that the PTX-loaded nanoparticles displayed significantly higher cytotoxicity to MCF-7 cells due to the sustained drug release manner. In short, the nanoparticles of the star-shaped copolymer CA-PLA-TPGS were able to achieve better therapeutic effects than those of the linear copolymer PLA-TPGS.

**Table 2:** **IC**_**50 **_**values of PTX formulations of Taxol^®^, PLA-TPGS nanoparticles, and CA-PLA-TPGS nanoparticles on MCF-7 cells (** ***n *** **= 6)**

**Incubation time (h)**	**IC**_**50 **_**(μg/mL)**		
	**Taxol^®^**	**PLA-TPGS NPs**	**CA-PLA-TPGS NPs**
24	45.47	49.20	46.63
48	38.13	35.41	34.71
72	28.32	27.40	15.22

### Animal studies

The advantages of PTX-loaded star-shaped CA-PLA-TPGS nanoparticles in breast cancer therapy were further confirmed in an animal model. In the present study, SCID mice bearing xenografts of a human breast carcinoma cell line were used to investigate the *in vivo* therapeutic effects of the star-shaped CA-PLA-TPGS nanoparticle formulation of PTX vs. Taxol^®^. The PTX-loaded CA-PLA-TPGS nanoparticle formulation was injected into the tumor every 4 days for three consecutive cycles. The tumor volume of the mice was monitored every 2 days until the 12th day, which was performed in comparison with the animal treated with Taxol^®^. Animals injected with vehicle (physiological saline, 0.9% NaCl) served as control.

**Figure 9:**
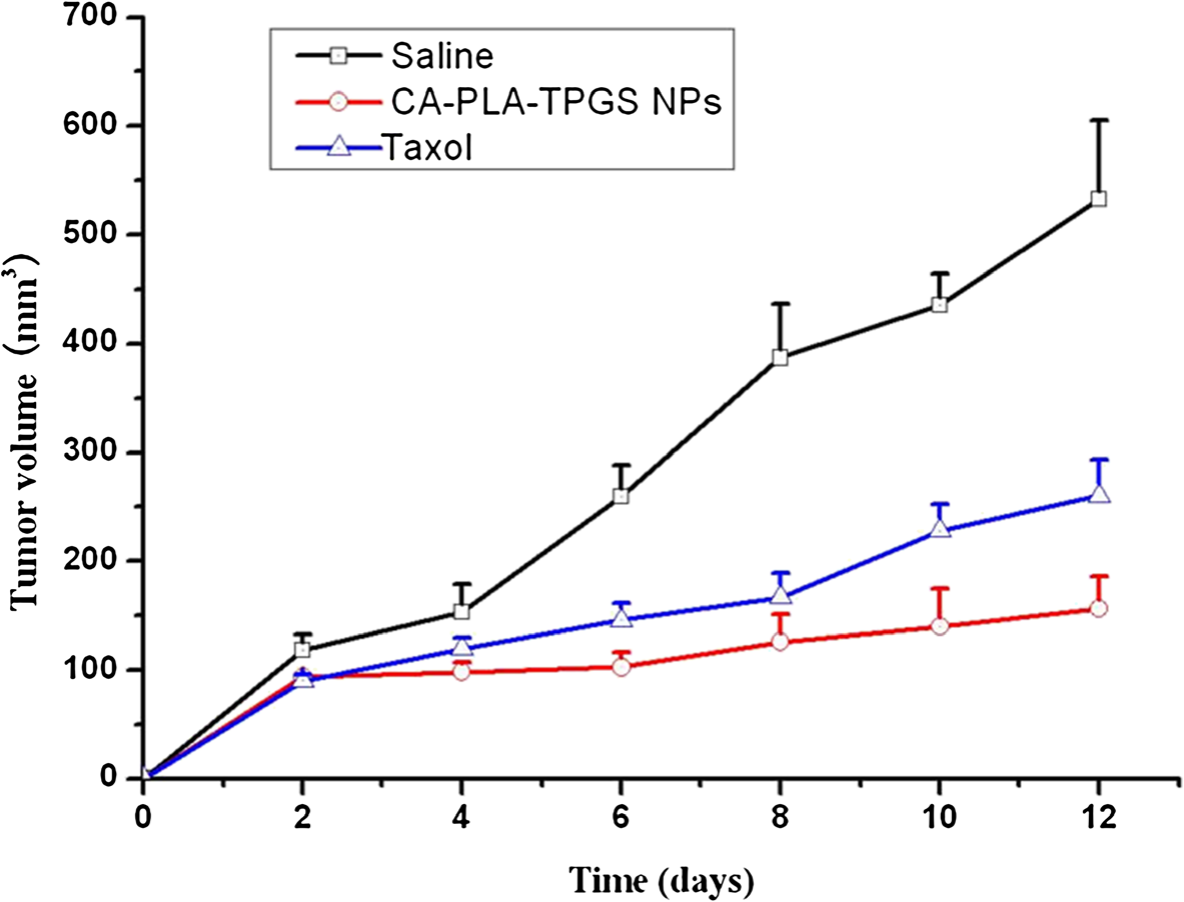
**Tumor growth curve of the mice after injection of the PTX-loaded CA-PLA-TPGS nanoparticles, Taxol^®^, and saline (** ***n = 5*).**

Figure 9 shows the tumor growth surveyed for 12 days in the mice after the intra-tumoral injection of the PTX-loaded CA-PLA-TPGS nanoparticles, Taxol^®^, and saline. It can be seen from this figure that the tumor size of the control group showed a statistically significant increase during the experimental period. However, the tumor growth of the groups treated with Taxol^®^ and the PTX-loaded star-shaped CA-PLA-TPGS nanoparticles was inhibited significantly. The tumor growth followed the order CA-PLA-TPGS nanoparticle treatment < Taxol^®^ < saline. In conclusion, such nanoparticles of star-shaped cholic acid-core PLA-TPGS block copolymer could be considered as a potentially promising and effective strategy for breast cancer treatment.

## Conclusions

A novel carrier system of star-shaped CA-PLA-TPGS nanoparticles for sustained and controlled delivery of paclitaxel for breast cancer treatment was developed in this research, which was compared with drug-loaded linear PLGA nanoparticles and linear PLA-TPGS copolymer nanoparticles. The three nanoparticle formulations were fabricated by a modified nanoprecipitation procedure. The particle size of the PTX-loaded star-shaped CA-PLA-TPGS nanoparticles could be prepared favorably approximately 120 nm in diameter. The star-shaped CA-PLA-TPGS nanoparticles could achieve higher drug loading content and entrapment efficiency, resulting in faster drug release as well as higher cellular uptake and cytotoxicity than the linear PLGA nanoparticles and the linear PLA-TPGS nanoparticles. The drug-loaded CA-PLA-TPGS nanoparticles were found to be stable, showing no change in the particle size and surface charge during 90-day storage of the aqueous solution. The *in vivo* cell studies indicated that the PTX-loaded star-shaped CA-PLA-TPGS nanoparticles were able to show significantly superior antitumor activity than the linear PLA-TPGS nanoparticle formulation and the clinical formulation Taxol^®^. In this research, the great advantages of such star-shaped CA-PLA-TPGS nanoparticles for paclitaxel formulation for breast cancer treatment were reported, which can also be used to other drugs of difficulty in formulation owing to high hydrophobicity.

## Competing interests

The authors declare that they have no competing interests.

## Authors’ contributions

XLT carried out the polymer synthesis, nanoparticle preparation, and cell studies. SYC carried out the polymer characterization and nanoparticle characterization. RBZ participated in the polymer synthesis and characterization. PL participated in the cell studies. HBC participated in the animal studies. LLS carried out the *in vivo* studies and participated in the design of the study. YZ conceived of the study and participated in its design and coordination. All authors read and approved the final manuscript.

## References

[B1] Siegel R Naishadham D Jemal A Cancer statistics, 2012 CA Cancer J Clin 2012 8 1 10 29 10.3322/caac.20138 22237781

[B2] Allen TM Cullis PR Drug delivery systems: entering the mainstream Science 2004 8 1818 1822 10.1126/science.1095833 15031496

[B3] Vivero-Escoto JL Slowing II Lin VS Tuning the cellular uptake and cytotoxicity properties of oligonucleotide intercalator-functionalized mesoporous silica nanoparticles with human cervical cancer cells MCF-7 Biomaterials 2012 8 1325 1333 10.1016/j.biomaterials.2009.11.009 19932923

[B4] Chen MC Sonaje K Chen KJ Sung HW A review of the prospects for polymeric nanoparticle platforms in oral insulin delivery Biomaterials 2011 8 9826 9838 10.1016/j.biomaterials.2011.08.087 21925726

[B5] Park S Kang S Chen X Kim EJ Kim J Kim N Kim J Jin MM Tumor suppression via paclitaxel-loaded drug carriers that target inflammation marker upregulated in tumor vasculature and macrophages Biomaterials 2013 8 598 605 10.1016/j.biomaterials.2012.10.004 23099063

[B6] Liu Q Li R Zhu Z Qian X Guan W Yu L Yang M Jiang X Liu B Enhanced antitumor efficacy, biodistribution and penetration of docetaxel-loaded biodegradable nanoparticles Int J Pharm 2012 8 350 358 10.1016/j.ijpharm.2012.04.008 22525076

[B7] Sonaje K Lin YH Juang JH Wey SP Chen CT Sung HW *In vivo* evaluation of safety and efficacy of self-assembled nanoparticles for oral insulin delivery Biomaterials 2009 8 2329 2339 10.1016/j.biomaterials.2008.12.066 19176244

[B8] Tomasina J Lheureux S Gauduchon P Rault S Malzert-Fréon A Nanocarriers for the targeted treatment of ovarian cancers Biomaterials 2013 8 1073 1101 10.1016/j.biomaterials.2012.10.055 23174141

[B9] Zeng X Tao W Mei L Huang L Tan C Feng SS Cholic acid-functionalized nanoparticles of star-shaped PLGA-vitamin E TPGS copolymer for docetaxel delivery to cervical cancer Biomaterials 2013 8 25 6058 6067 10.1016/j.biomaterials.2013.04.052 23694904

[B10] Mi Y Liu XL Zhao J Ding J Feng SS Multimodality treatment of cancer with herceptin conjugated, thermomagnetic iron oxides and docetaxel loaded nanoparticles of biodegradable polymers Biomaterials 2012 8 7519 7529 10.1016/j.biomaterials.2012.06.100 22809649

[B11] Thamake SI Raut SL Gryczynski Z Ranjan AP Vishwanatha JK Alendronate coated poly-lactic-co-glycolic acid (PLGA) nanoparticles for active targeting of metastatic breast cancer Biomaterials 2012 8 7164 7173 10.1016/j.biomaterials.2012.06.026 22795543

[B12] Park W Kim DG Kang HC Bae YH Na K Multi-arm histidine copolymer for controlled release of insulin from poly(lactide-co-glycolide) microsphere Biomaterials 2012 8 8848 8857 10.1016/j.biomaterials.2012.08.042 22959184

[B13] Yang C Jiang L Bu S Zhang L Xie X Zeng Q Zhu D Zheng Y Intravitreal administration of dexamethasone-loaded PLGA-TPGS nanoparticles for the treatment of posterior segment diseases J Biomed Nanotechnol 2013 8 9 1617 1623 10.1166/jbn.2013.1646 23980509

[B14] Fox ME Szoka FC Frechet AMJ Soluble polymer carriers for the treatment of cancer: the importance of molecular architecture Acc Chem Res 2009 8 1141 1151 10.1021/ar900035f 19555070 PMC2759385

[B15] Cuon NV Li YL Hsieh MF Targeted delivery of doxorubicin to human breast cancers by folate-decorated star-shaped PEG–PCL micelle J Mater Chem 2012 8 1006 1020 10.1039/c1jm13588k

[B16] Zhang ZP Tan SW Feng SS Vitamin E TPGS as a molecular biomaterial for drug delivery Biomaterials 2012 8 4889 4906 10.1016/j.biomaterials.2012.03.046 22498300

[B17] Zhang ZP Mei L Feng SS Vitamin E d-a-tocopheryl polyethylene glycol 1000 succinate-based nanomedicine Nanomedicine 2012 8 1645 1647 10.2217/nnm.12.117 23210711

[B18] Li ZB Kesselman E Talmon Y Hillmyer MA Lodge TP Multicompartment micelles from ABC miktoarm stars in water Science 2004 8 98 101 10.1126/science.1103350 15459387

[B19] Lapienis G Star-shaped polymers having PEO arms Prog Polym Sci 2009 8 852 892 10.1016/j.progpolymsci.2009.04.006

[B20] Ouyang CP Liu Q Zhao SX Ma GL Zhang ZP Song CX Synthesis and characterization of star-shaped poly(lactide-*co*-glycolide) and its drug-loaded microspheres Polym Bull 2012 8 27 36 10.1007/s00289-011-0516-x

[B21] Zhang X Cheng J Wang Q Zhong Z Zhuo R Miktoarm copolymers bearing one poly(ethylene glycol) chain and several poly(ϵ-caprolactone) chains on a hyperbranched polyglycerol core Macromolecules 2010 8 6671 6677 10.1021/ma100653u

[B22] Maglio G Nese G Nuzzo M Palumbo R Synthesis and characterization of star-shaped diblock poly(ϵ-caprolactone)/poly(ethylene oxide) copolymers Macromol Rapid Commun 2004 8 1139 1144 10.1002/marc.200400113

[B23] Lapienis G Functionalized star-shaped polymers having PEO and/or polyglycidyl arms and their properties Polymer 2009 8 77 84 10.1016/j.polymer.2008.10.034

[B24] Nabid MR Rezaei SJT Sedghi R Niknejad H Entezami AA Oskooie HA Heravi MM Self-assembled micelles of well-defined pentaerythritol-centered amphiphilic A4B8 star-block copolymers based on PCL and PEG for hydrophobic drug delivery Polymer 2011 8 2799 2809 10.1016/j.polymer.2011.04.054

[B25] Koyama Y Ito T Kimura T Murakami A Yamaoka T Effect of cholesteryl side chain and complexing with cholic acid on gene transfection by cationic poly(ethylene glycol) derivatives J Control Release 2001 8 357 364 10.1016/S0168-3659(01)00521-1 11733102

[B26] Mehnert W Mäder K Solid lipid nanoparticles, production, characterization and applications Adv Drug Delivery Rev 2012 8 83 101 10.1016/s0169-409x(01)00105-3 11311991

[B27] Mei L Zhang Y Zheng Y Tian G Song CX Yang DY Chen HL Sun HF Tian Y Liu K Li Z Huang L A novel paclitaxel-loaded poly(ϵ-caprolactone)/pluronic F68 nanoparticle overcoming multidrug resistance for breast cancer treatment Nanoscale Res Lett 2009 8 1530 1539 10.1007/s11671-009-9431-6 20652101 PMC2894322

[B28] Ma YD Huang LQ Song CX Zeng XW Liu G Mei L Nanoparticle formulation of poly(*ϵ*-caprolactone-co-lactide)-d-*α*-tocopheryl polyethylene glycol 1000 succinate random copolymer for breast cancer treatment Polymer 2010 8 5952 5959 10.1016/j.polymer.2010.10.029

[B29] Guo J Gao X Su L Xia H Gu G Pang Z Jiang X Yao L Chen J Chen H Aptamer-functionalized PEG-PLGA nanoparticles for enhanced anti-glioma drug delivery Biomaterials 2011 8 8010 8020 10.1016/j.biomaterials.2011.07.004 21788069

[B30] Zhu Z Li Y Li X Li R Jia Z Liu B Guo W Wu W Jiang X Paclitaxel-loaded poly(*N*-vinylpyrrolidone)-*b*-poly(ϵ-caprolactone) nanoparticles: preparation and antitumor activity *in vivo* J Control Release 2010 8 438 446 10.1016/j.jconrel.2009.11.002 19896997

[B31] Schubert S Delaney JT Schubert US Nanoprecipitation and nanoformulation of polymers: from history to powerful possibilities beyond poly(lactic acid) Soft Matter 2011 8 1581 1588 10.1039/c0sm00862a

[B32] Perrault SD Walkey C Jennings T Fischer HC Chan WC Mediating tumor targeting efficiency of nanoparticles through design Nano Lett 2009 8 1909 1915 10.1021/nl900031y 19344179

[B33] Yan F Zhang C Zheng Y Mei L Tang L Song C Sun H Huang L The effect of poloxamer 188 on nanoparticle morphology, size, cancer cell uptake, and cytotoxicity Nanomedicine 2010 8 170 178 10.1016/j.nano.2009.05.004 19447200

[B34] Fang C Bhattarai N Sun C Zhang M Functionalized nanoparticles with long-term stability in biological media Small 2009 8 14 1637 1641 10.1002/smll.200801647 19334014 PMC2883049

[B35] Ma Y Zheng Y Zeng X Jiang L Chen H Liu R Huang L Mei L Novel paclitaxel-loaded nanoparticles based on PCL-Tween 80 copolymer for cancer treatment Int J Nanomedicine 2011 8 2679 2688 22114498 10.2147/IJN.S25251 PMC3218581

[B36] Muthu MS Kulkarni SA Raju A Feng SS Theranostic liposomes of TPGS coating for targeted co-delivery of paclitaxel and quantum dots Biomaterials 2012 8 3494 3501 10.1016/j.biomaterials.2012.01.036 22306020

[B37] Baimark Y Srisa-ard M Preparation of drug-loaded microspheres of linear and star-shaped poly(D, L-lactide)s and their drug release behaviors J Appl Polym Sci 2012 8 3871 3878 10.1002/app.35473

[B38] Chen H Zheng Y Tian G Tian Y Zeng X Liu G Liu K Li L Li Z Mei L Huang L Oral delivery of DMAB-modified docetaxel-loaded PLGA-TPGS nanoparticles for cancer chemotherapy Nanoscale Res Lett 2011 8 4 27502629 10.1007/s11671-010-9741-8 PMC3102336

[B39] Feng SS Mei L Anitha P Gan CW Zhou WY Poly(lactide)–vitamin E derivative/montmorillonite nanoparticle formulations for the oral delivery of paclitaxel Biomaterials 2009 8 3297 3306 10.1016/j.biomaterials.2009.02.045 19299012

[B40] Tao W Zeng X Liu T Wang Z Xiong Q Ouyang C Huang L Mei L Docetaxel-loaded nanoparticles based on star-shaped mannitol-core PLGA-TPGS diblock copolymer for breast cancer therapy Acta Biomater 2013 8 8910 8920 10.1016/j.actbio.2013.06.034 23816645

[B41] Rejman J Oberle V Zuhorn IS Hoekstra D Size-dependent internalization of particle via the pathways of clathrin- and caveolae-mediated endocytosis Biochem J 2004 8 159 169 10.1042/BJ20031253 14505488 PMC1223843

[B42] Zhang Y Tang L Sun L Bao J Song C Huang L Liu K Tian Y Tian G Li Z Sun H Mei L A novel paclitaxel-loaded poly(ϵ-caprolactone)/Poloxamer 188 blend nanoparticle overcoming multidrug resistance for cancer treatment Acta Biomater 2010 8 2045 2052 10.1016/j.actbio.2009.11.035 19969111

[B43] Wang J Sun J Chen Q Gao Y Li L Li H Leng D Wang Y Sun Y Jing Y Wang S He Z Star-shape copolymer of lysine-linked di-tocopherol polyethylene glycol 2000 succinate for doxorubicin delivery with reversal of multidrug resistance Biomaterials 2012 8 6877 6888 10.1016/j.biomaterials.2012.06.019 22770799

[B44] Zheng Y Chen H Zeng X Liu Z Xiao X Zhu Y Gu D Mei L Surface modification of TPGS-*b*-(PCL-*ran*-PGA) nanoparticles with polyethyleneimine as a co-delivery system of TRAIL and endostatin for cervical cancer gene therapy Nanoscale Res Lett 2013 8 1 161 10.1186/1556-276X-8-161 23570619 PMC3639870

[B45] Qiu B Ji M Song X Zhu Y Wang Z Zhang X Wu S Chen H Mei L Zheng Y Co-delivery of docetaxel and endostatin by a biodegradable nanoparticle for the synergistic treatment of cervical cancer Nanoscale Res Lett 2012 8 1 666 10.1186/1556-276X-7-666 23216701 PMC3598810

